# Crystal structure of penta­sodium hydrogen dicitrate from synchrotron X-ray powder diffraction data and DFT comparison

**DOI:** 10.1107/S2056989017001256

**Published:** 2017-01-27

**Authors:** Alagappa Rammohan, James A. Kaduk

**Affiliations:** a Atlantic International University, Honolulu HI, USA; b Illinois Institute of Technology, Chicago IL, USA

**Keywords:** powder diffraction, crystal structure, density functional theory, citrate, sodium

## Abstract

The crystal structure of penta­sodium hydrogen dicitrate, Na_5_H(C_6_H_5_O_7_)_2_, has been solved and refined using synchrotron X-ray powder diffraction data, and optimized using density functional techniques. Each of the two independent citrate anions is joined into a dimer by very strong centrosymmetric O—H⋯O hydrogen bonds, with O⋯O distances of 2.419 and 2.409 Å. Four octa­hedrally coordinated Na^+^ ions share edges to form open layers parallel to the *ab* plane. A fifth Na^+^ ion in trigonal–bipyramidal coordination shares faces with NaO_6_ octahedra on both sides of these layers.

## Abstract 2

The crystal structure of penta­sodium hydrogen dicitrate has been solved and refined using synchrotron X-ray powder diffraction data, and optimized using density functional techniques.

## Chemical context   

In the course of a systematic study of the crystal structures of Group 1 (alkali metal) citrate salts to understand the anion’s conformational flexibility, ionization, coordination tendencies, and hydrogen bonding, we have determined several new crystal structures. Most of the new structures were solved using X-ray powder diffraction data (laboratory and/or synchrotron), but single crystals were used where available. The general trends and conclusions about the sixteen new compounds and twelve previously characterized structures are being reported separately (Rammohan & Kaduk, 2017*a* ▸). Nine of the new structures – NaKHC_6_H_5_O_7_, NaK_2_C_6_H_5_O_7_, Na_3_C_6_H_5_O_7_, NaH_2_C_6_H_5_O_7_, Na_2_HC_6_H_5_O_7_, K_3_C_6_H_5_O_7_, Rb_2_HC_6_H_5_O_7_, Rb_3_C_6_H_5_O_7_(H_2_O), and Rb_3_C_6_H_5_O_7_ – have been published recently (Rammohan & Kaduk, 2016*a* ▸,*b* ▸,*c* ▸,*d* ▸,*e* ▸, 2017*b* ▸,*c* ▸,*d* ▸; Rammohan *et al.*, 2016 ▸), and two additional structures – KH_2_C_6_H_5_O_7_ and KH_2_C_6_H_5_O_7_(H_2_O)_2_ – have been communicated to the CSD (Kaduk & Stern, 2016*a* ▸,*b* ▸).
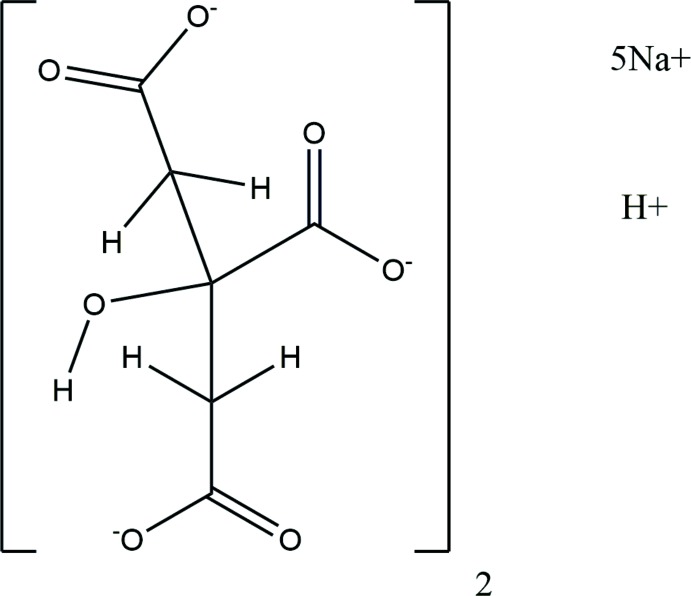


## Structural commentary   

**Figure 1:**
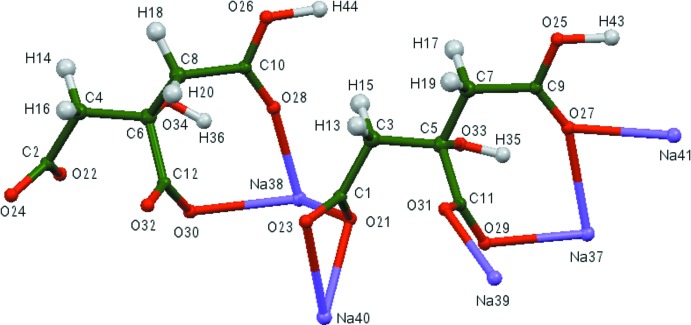
The asymmetric unit of Na_5_H(C_6_H_5_O_7_)_2_, with the atom numbering. The atoms are represented by 50% probability spheroids.

**Figure 2:**
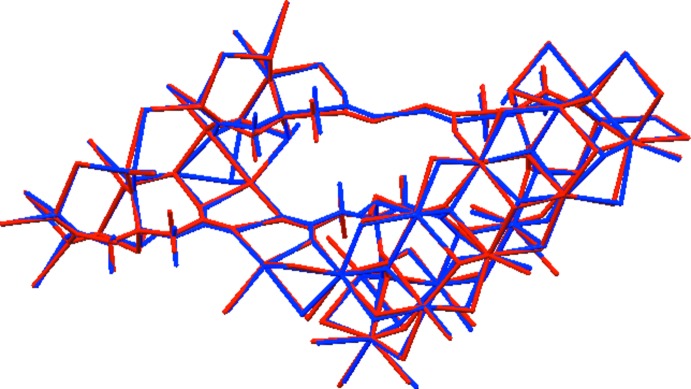
Comparison of the refined and optimized structures of penta­sodium hydrogen dicitrate. The refined structure is in red, and the DFT-optimized structure is in blue.

The compound Na_5_H(C_6_H_5_O_7_)_2_ was unexpectedly synthesized by heating Na_2_HC_6_H_5_O_7_(H_2_O)_1.5_. The asymmetric unit of the title compound is shown in Fig. 1 ▸. The root-mean-square deviation of the non-hydrogen atoms in the Rietveld-refined and DFT-optimized structures is 0.216 Å (Fig. 2 ▸). The reasonable agreement between the two structures is evidence that the experimental structure is correct (van de Streek & Neumann, 2014 ▸). This discussion uses the DFT-optimized structure. Most of the bond lengths, bond angles, and torsion angles fall within the normal ranges indicated by a *Mercury* Mogul geometry check (Macrae *et al.*, 2008 ▸). Both the O28—C10 bond length of 1.249 Å [*Z*-score = 3.4; average = 1.213 (13) Å] and the O28—C10—C8 angle of 120.4° [*Z*-score = 4.0; average = 126.9 (16)°] are flagged as unusual. Since this oxygen atom also coordinates to an Na^+^ cation, it is not unreasonable to encounter some slightly unusual geometry. Each Na^+^ cation is chelated by at least one citrate anion. The chelation modes include hydrox­yl/terminal carboxyl, terminal/central carboxyl, and coordination of both oxygen atoms of a terminal carboxyl group.

**Figure 3:**
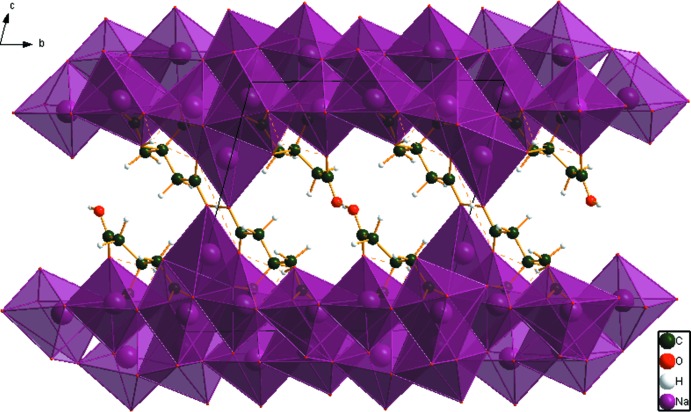
Crystal structure of Na_5_H(C_6_H_5_O_7_)_2_, viewed down the *a* axis.

The structure contains five independent Na^+^ cations. Na37, Na38, Na39, and Na40 exhibit octahedral coordination spheres, with bond-valence sums of 1.19, 1.24, 1.04, and 1.17 valence units, respectively. Na41 is only five-coordinate with a trigonal–bipyramidal coordination sphere, but its bond-valence sum is 1.26. The only O atom not coordinating to an Na^+^ cation is O26, which participates in very strong centrosymmetric hydrogen bonds. The octahedra involving Na37–Na40 share edges to form open layers parallel to the *ab* plane. Trigonal–bipyramidal Na41O_5_ polyhedra share faces with Na37O_6_ and Na39O_6_ octahedra on both sides of these layers. The crystal structure is illustrated in Fig. 3 ▸.

The Mulliken overlap populations and atomic charges indicate that the metal–oxygen bonding is ionic. Comparison of the structures of the starting material Na_2_HC_6_H_5_O_7_(H_2_O)_1.5_ and the title compound does not suggest any plausible mechanism for the conversion.

The Bravais–Friedel–Donnay–Harker (Bravais, 1866 ▸; Friedel, 1907 ▸; Donnay & Harker, 1937 ▸) morphology suggests that we might expect elongated morphology for penta­sodium hydrogen dicitrate, with {100} as the principal axis. A 4th-order spherical harmonic texture model was included in the refinement. The texture index was 1.014, indicating that preferred orientation was slight for this rotated capillary specimen.

## Supra­molecular features   

The layers are connected by very strong centrosymmetric O26—H44⋯O26 and O25—H43⋯O25 hydrogen bonds (Table 1 ▸). The O26⋯O26 distance is 2.419 Å and the O25⋯O25 distance is 2.409 Å, making these among the shortest hydrogen bonds. The Mulliken overlap populations in the hydrogen bonds are 0.145 and 0.136 *e*, which correspond to 20.8 and 20.2 kcal mol^−1^ for each hydrogen bond (Rammohan & Kaduk, 2017*a* ▸). These hydrogen bonds link two citrates into dimers.

The Mulliken overlap populations indicate that the hydroxyl groups O33—H35 and O34—H36 each act as donors in three hydrogen bonds. One [with graph set *S*(5)] is to the central carboxyl­ate group, and another is intra­molecular to a terminal carboxyl group. The third hydrogen bond is inter­molecular. These hydrogen bonds are much weaker than the centrosymmetric ones, contributing 5–8 kcal mol^−1^ to the crystal energy.

## Database survey   

Details of the comprehensive literature search for citrate structures are presented in Rammohan & Kaduk (2017*a* ▸). A reduced cell search of the cell of penta­sodium hydrogen dicitrate in the Cambridge Structural Database (Groom *et al.*, 2016 ▸) (increasing the default tolerance from 1.5 to 2.0%) yielded 98 hits, but combining the cell search with the elements C, H, Na, and O only yielded no hits.

## Synthesis and crystallization   

The title compound was prepared by heating commercial reagent Na_2_HC_6_H_5_O_7_(H_2_O)_1.5_ (Sigma–Aldrich lot BCBC6031V) from 295–453K at 14 K min^−1^ in air. The crystal structure of this reagent is reported in Rammohan *et al.* (2016 ▸). After holding at 453 K for two minutes, the sample began to discolour (turn yellow), and it was taken from the oven and sealed in a glass jar to cool.

## Refinement details   

**Figure 4:**
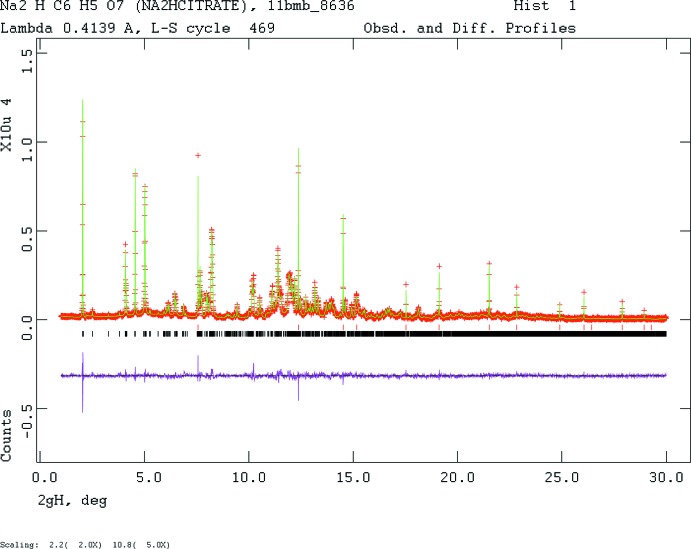
Rietveld plot for the refinement of Na_5_H(C_6_H_5_O_7_)_2_. The red crosses represent the observed data points, and the green line is the calculated pattern. The magenta curve is the difference pattern, plotted at the same scale as the other patterns. The vertical scale has been multiplied by a factor of 2.0 at 2.2°, and by 5.0 at 10.8°. The row of black tick marks indicates the reflection positions. The red tick marks indicate the positions of the peaks of the Si inter­nal standard.

Crystal data, data collection and structure refinement details are summarized in Table 2 ▸. The sample was blended with NIST SRM 640b silicon inter­nal standard in a Spex 8000 mixer/mill, and packed into a standard sample holder. It was protected from the atmosphere by a thin Kapton window attached to the cell edges with Vaseline. The pattern was measured on a Bruker D2 Phaser at IIT, and eventually at 11-BM at APS/ANL (Lee *et al.*, 2008 ▸; Wang *et al.*, 2008 ▸). The structure was solved and refined using the synchrotron data. Diffraction data are displayed in Fig. 4 ▸.

The synchrotron pattern was indexed with *Jade 9.5* (MDI, 2012 ▸) on a primitive triclinic unit cell having *a* = 6.263, *b* = 12.029, *c* = 12.132 Å, α = 74.145, β = 81.530, γ = 80.8 6°, and *V* = 863.06 Å^3^. The volume corresponds to four citrate anions per cell. A Le Bail fit using this cell yielded a reduced χ^2^ = 2.866, but it was not possible to solve the crystal structure using this unit cell.

Removing the weak peak at 2.510° yielded a new cell, with *a* = 6.131, *b* = 6.352, *c* = 12.142 Å, α = 100.486, β = 98.839, γ = 110.4 0°, and *V* = 435.852 Å^3^. This is a sub-cell of the original cell. The volume corresponds to two citrate anions per cell, and space group *P* 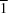 was assumed. A Le Bail fit yielded a reduced χ^2^ of 2.716.

The structure was solved in the sub-cell using *DASH* 3.3.2 (David *et al.*, 2006 ▸), with a citrate anion and two Na^+^ cations as fragments. Two of the 25 simulated annealing runs yielded figures of merit much lower than the rest. Since two O13 atoms were 2.53 Å apart (related by a centre of symmetry), a hydrogen was placed at the centre. Refinement of this model yielded a reduced χ^2^ of 2.6, but the charge did not balance. A difference-Fourier map indicated a peak 2.33 Å from O13; this is too close to be an oxygen atom, but is reasonable for an Na atom. Refinement of this model yielded a reduced χ^2^ of 1.85, and an Na occupancy of 1/2.

The structure was transformed (matrix [0
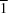
0/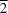 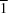
0/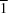 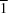 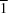]) to the original cell using *Materials Studio* (Dassault Systemes, 2014 ▸). The occupancies of the now two half-Na were refined. They refined to 1/0, and the low-occupancy Na moved too close to other atoms. Refinement of the resulting Na_5_H(citrate)_2_ model yielded a reduced χ^2^ of 1.829. This larger cell accounts for the 2.510° peak and several other very weak peaks not explained by the sub-cell. A possible *C*-centering, as suggested by *PLATON* (Spek, 2009 ▸), is not present.

Pseudo-Voigt profile coefficients were as parameterized in Thompson *et al.* (1987 ▸) with profile coefficients for Simpson’s rule integration of the pseudo-Voigt function according to Howard (1982 ▸). The asymmetry correction of Finger *et al.* (1994 ▸) was applied, and microstrain broadening by Stephens (1999 ▸). The structure was refined by the Rietveld method using *GSAS/EXPGUI* (Larson & Von Dreele, 2004 ▸; Toby, 2001 ▸). All C—C and C—O bond lengths were restrained, as were all bond angles. The C—C bonds between the terminal carboxyl carbon atoms and the adjacent carbon atoms were restrained at 1.51 (1) Å, the C—C bonds between the central carbon atoms and the adjacent carbon atoms at 1.54 (1) Å, the C—C bond between the central carbon atom and the central carboxyl carbon at 1.55 (1) Å, the C—O bond to the hydroxyl group at 1.42 (3) Å, and the C—O bonds in the carboxyl­ate groups at 1.27 (3) Å. The tetra­hedral carbon bond angles were restrained at 109 (3)°, and the angles in the planar carboxyl groups at 120 (3)°. The hydrogen atoms were included at fixed positions, which were recalculated during the course of the refinement using *Materials Studio* (Dassault Systemes, 2014 ▸). The *U*_iso_ values of the C and O atoms were constrained to be equal, and the *U*_iso_ values of the hydrogen atoms were constrained to be 1.3 times those of the atoms to which they are attached. A common *U*_iso_ value was refined for the Na atoms.

## DFT calculations   

A density functional geometry optimization (fixed experimental unit cell) was carried out using *CRYSTAL09* (Dovesi *et al.*, 2005 ▸). The basis sets for the C, H, and O atoms were those of Gatti *et al.* (1994 ▸), and the basis set for Na was that of Dovesi *et al.* (1991 ▸). The calculation used 8 *k*-points and the B3LYP functional, and took about eight days on a 2.4 GHz PC. *U*_iso_ values were assigned to the optimized fractional coordinates based on the *U*_eq_ from the refined structure.

## Supplementary Material

Crystal structure: contains datablock(s) NA2HCITRATE_2_publ, Na5Hcit2_DFT, NA2HCITRATE_2_overall, NA2HCITRATE_2_phase_1, NA2HCITRATE_2_phase_2, NA2HCITRATE_2_p_01. DOI: 10.1107/S2056989017001256/vn2125sup1.cif

Supporting information file. DOI: 10.1107/S2056989017001256/vn2125NA2HCITRATE_2_phase_2sup2.cml

CCDC references: 1529603, 1529604, 1529605

Additional supporting information:  crystallographic information; 3D view; checkCIF report

## Figures and Tables

**Table 1:** Hydrogen-bond geometry (Å, °) for Na5Hcit2_DFT[Chem scheme1]

*D*—H⋯*A*	*D*—H	H⋯*A*	*D*⋯*A*	*D*—H⋯*A*
O26—H44⋯O26^i^	1.210	1.210	2.419	180.0
O25—H43⋯O25^ii^	1.205	1.205	2.409	180.0
O33—H35⋯O23^iii^	0.975	2.045	2.799	132.8
O33—H35⋯O29	0.975	2.450	2.664	91.7
O34—H36⋯O30	0.971	2.212	2.631	104.6
O34—H36⋯O28	0.971	2.302	2.831	113.4
O34—H36⋯O24^iii^	0.971	2.385	3.223	144.3

Symmetry codes: (i) 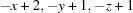; (ii) 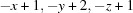; (iii) 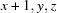.

**Table 2:** Experimental details

Crystal data	
Chemical formula	Na_5_H(C_6_H_5_O_7_)_2_
*M*_r_	494.16
Crystal system, space group	Triclinic, *P* 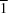
Temperature (K)	295
*a*, *b*, *c* (Å)	6.35262 (9), 11.98628 (18), 12.16544 (16)
α, β, γ (°)	73.8374 (13), 80.8808 (15), 80.7103 (10)
*V* (Å^3^)	871.72 (2)
*Z*	2
Radiation type	Synchrotron, λ = 0.413891 Å
Specimen shape, size (mm)	Cylinder, 1.5 × 1.5

Data collection	
Diffractometer	11-BM APS
Specimen mounting	Kapton capillary
Data collection mode	Transmission
Scan method	Step
*T*_min_, *T*_max_	1.000, 1.000
2θ values (°)	2θ_min_ = 0.5 2θ_max_ = 50.0 2θ_step_ = 0.001

Refinement	
*R* factors and goodness of fit	*R*_p_ = 0.083, *R*_wp_ = 0.097, *R*_exp_ = 0.073, *R*(*F* ^2^) = 0.085, χ^2^ = 1.823
No. of parameters	145
No. of restraints	58

The same symmetry and lattice parameters were used for the DFT calculation. Computer programs: *DIFFRAC.Measurement* (Bruker, 2009 ▸), *DASH* (David *et al.*, 2006 ▸), *GSAS* (Larson & Von Dreele, 2004 ▸), *DIAMOND* (Crystal Impact, 2015 ▸) and *publCIF* (Westrip, 2010 ▸).

## supplementary crystallographic information

### Crystal data

**Table:**

Na_5_H(C_6_H_5_O_7_)_2_	α = 73.8374°
*M**_r_* = 494.13	β = 80.8808°
Triclinic, *P*1	γ = 80.7103°
Hall symbol: -P 1	*V* = 871.72 Å^3^
*a* = 6.3526 Å	*Z* = 2
*b* = 11.9863 Å	*D*_x_ = 1.883 Mg m^−^^3^
*c* = 12.1654 Å	*T* = 295 K

### Data collection

**Table:**

Density functional calculation	*k* = →
*h* = →	*l* = →

### Fractional atomic coordinates and isotropic or equivalent isotropic displacement parameters (Å^2^)

**Table:**

	*x*	*y*	*z*	*U*_iso_*/*U*_eq_	
C1	−0.21093	0.58380	0.83744	0.01060*	
C2	0.28547	0.08699	0.82748	0.01060*	
C3	−0.11904	0.65713	0.72068	0.01600*	
C4	0.34300	0.18316	0.71790	0.01600*	
C5	0.06950	0.72103	0.72811	0.01600*	
C6	0.53786	0.24180	0.72599	0.01600*	
C7	0.11665	0.81156	0.61196	0.01600*	
C8	0.55181	0.35732	0.63154	0.01600*	
C9	0.29966	0.88149	0.60472	0.01060*	
C10	0.77028	0.40070	0.61710	0.01060*	
C11	0.00917	0.77940	0.83069	0.01060*	
C12	0.50875	0.26301	0.84778	0.01060*	
O21	−0.08985	0.50169	0.89446	0.01060*	
O22	0.43125	0.00445	0.86367	0.01060*	
O23	−0.40674	0.61148	0.87458	0.01060*	
O24	0.09817	0.09733	0.88046	0.01060*	
O25	0.34064	0.95546	0.50492	0.01060*	
O26	0.82824	0.46330	0.51541	0.01060*	
O27	0.40445	0.86921	0.68646	0.01060*	
O28	0.87727	0.37570	0.70033	0.01060*	
O29	0.11832	0.74256	0.91351	0.01060*	
O30	0.65064	0.21054	0.91295	0.01060*	
O31	−0.15265	0.85830	0.82412	0.01060*	
O32	0.34170	0.32615	0.87533	0.01060*	
O33	0.25360	0.63635	0.74506	0.01060*	
O34	0.72908	0.16202	0.71125	0.01060*	
H35	0.36530	0.67271	0.76366	0.01380*	
H36	0.83443	0.18009	0.75138	0.01380*	
Na37	0.46548	0.79769	0.89886	0.01270*	
Na38	0.97393	0.30775	0.88552	0.01270*	
Na39	0.22004	0.98848	0.06887	0.01270*	
Na40	0.71654	0.49717	0.07535	0.01270*	
Na41	0.66889	0.98575	0.69266	0.01270*	
H13	−0.24775	0.72123	0.68279	0.02090*	
H15	−0.05830	0.59958	0.66395	0.02090*	
H14	0.62099	0.85243	0.35789	0.02090*	
H16	0.79387	0.74936	0.29832	0.02090*	
H18	0.47857	0.65251	0.45109	0.02090*	
H20	0.57361	0.57501	0.34726	0.02090*	
H17	−0.16193	0.23620	0.45478	0.02090*	
H19	0.02421	0.12681	0.41491	0.02090*	
H43	0.50000	1.00000	0.50000	0.01470*	
H44	1.00000	0.50000	0.50000	0.01470*	

### Bond lengths (Å)

**Table:**

C1—C3	1.529	C8—H18^i^	1.094
C1—O21	1.259	C8—H20^i^	1.096
C1—O23	1.278	C9—O25	1.305
C2—C4	1.538	C9—O27	1.245
C2—O22	1.273	C10—O26	1.287
C2—O24	1.265	C10—O28	1.249
C3—C5	1.549	C11—O29	1.251
C3—H13	1.091	C11—O31	1.276
C3—H15	1.094	C12—O30	1.266
C4—C6	1.547	C12—O32	1.257
C4—H14^i^	1.098	O25—H43	1.205
C4—H16^i^	1.090	O26—H44	1.210
C5—C7	1.543	O33—H35	0.975
C5—C11	1.563	O34—H36	0.971
C5—O33	1.420	H14—C4^i^	1.098
C6—C8	1.539	H16—C4^i^	1.090
C6—C12	1.550	H18—C8^i^	1.094
C6—O34	1.440	H20—C8^i^	1.096
C7—C9	1.518	H17—C7^ii^	1.096
C7—H17^ii^	1.096	H19—C7^ii^	1.095
C7—H19^ii^	1.095	H43—O25^iii^	1.205
C8—C10	1.527	H44—O26^iv^	1.210

Symmetry codes: (i) −*x*+1, −*y*+1, −*z*+1; (ii) −*x*, −*y*+1, −*z*+1; (iii) −*x*+1, −*y*+2, −*z*+1; (iv) −*x*+2, −*y*+1, −*z*+1.

### Hydrogen-bond geometry (Å, º)

**Table:**

*D*—H···*A*	*D*—H	H···*A*	*D*···*A*	*D*—H···*A*
O26—H44···O26^iv^	1.210	1.210	2.419	180.0
O25—H43···O25^iii^	1.205	1.205	2.409	180.0
O33—H35···O23^v^	0.975	2.045	2.799	132.8
O33—H35···O29	0.975	2.450	2.664	91.7
O34—H36···O30	0.971	2.212	2.631	104.6
O34—H36···O28	0.971	2.302	2.831	113.4
O34—H36···O24^v^	0.971	2.385	3.223	144.3

Symmetry codes: (iii) −*x*+1, −*y*+2, −*z*+1; (iv) −*x*+2, −*y*+1, −*z*+1; (v) *x*+1, *y*, *z*.
